# Horizontal and Vertical Distributions of Chromium in a Chromate Production District of South Central China

**DOI:** 10.3390/ijerph15040571

**Published:** 2018-03-22

**Authors:** Bin Zhou, Daoyou Huang, Jinshui Wu, Qihong Zhu, Hanhua Zhu

**Affiliations:** 1 Key Laboratory for Agro-Ecological Processes in Subtropical Region, Institute of Subtropical Agriculture, Chinese Academy of Sciences, Changsha 410125, China; bzhou@isa.ac.cn (B.Z.); jswu@isa.ac.cn (J.W.); qhzhu@isa.ac.cn (Q.Z.); hhzhu@isa.ac.cn (H.Z.); 2 University of Chinese Academy of Sciences, Beijing 100049, China; 3 Yonker Environmental Protection Company, Ltd., Changsha 410330, China

**Keywords:** chromate production site, chromium, horizontal and vertical distributions

## Abstract

To study the horizontal and vertical distribution of chromium (Cr) in the soil of a chromate production site (CPS) and its nearby area (NA-CPS) in south central China, 61 profiles (depth: 14 m) in the CPS and 69 samples (topsoil) were excavated following a grid-sampling method. The geographic coordinates, elevation, and types of soil layers were recorded, and the total Cr in the soil and the total Cr and Cr(VI) in the leachate of the soil and in the groundwater were determined. Migration of Cr in surface soils may be represented in terms of a multiple linear regression equation (*R*^2^_adj_ = 0.632). Distance, elevation, and pH are the primary factors that influence the horizontal distribution of Cr content in the surface soils, while the Cr concentration in different soil profiles mostly obeys the positive or negative binomial distributions. For a positive distribution, the Cr concentration decreases with increasing depth in the 0.0–8.0 m soil layer, under the fixing effect of soil. However, it shows an upward trend with a depth in the 8–14 m soil layer under the influence of Cr-polluted phreatic water. Under a negative distribution, Cr content is stable in the 0–6 m layer because of the influence of chromite ore processing residue mixed with miscellaneous fills, but it decreases obviously in the 6–14 m layer under the fixing effect of soil. Similar vertical distributions were observed for pH, *L_Cr_*, *L_Cr_*^6+^, and *P_Cr_*^6+^. The decreasing amplitude of the Cr concentration for binomial distributions is mainly affected by the Cr concentration, pH, and *LR_Cr_* of the soil. Moreover, *P_Cr_^6+^* of soil increases with pH, and the type of soil layer is the primary factor influencing *LR_Cr_* in the soil profiles. Our results of the horizontal and vertical distributions of Cr could be used to guide investigations that are focused on reducing the number of samples in the horizontal and vertical directions at CPSs, and to improve risk assessments of CPSs and nearby areas.

## 1. Introduction

As an important industrial metal element, chromium (Cr) is widely applied in the metallurgy, pigment production, electroplating, leather tanning, and antiseptics industries. Six million tons of chromite ore processing residues (COPRs) are generated annually during the process of chromate production in China and India [1]. The improper disposal of COPR has resulted in severe soil Cr pollution at chromate production sites (CPSs). Reports have shown that the reuse of COPR as a filling material has resulted in soil pollution by Cr(VI) at 15 sites in Glasgow (UK), where 10-m deep soil was polluted, and the highest Cr(VI) concentration was up to 16,000 mg/kg [2]. Soils and groundwater in many CPSs in China have been severely polluted by Cr(VI), because of the improper disposal of COPR [3]. Cr(VI) in CPSs can cause cancer and other genetic mutations [4]. Therefore, it is urgent to investigate, assess risk, and remediate the soils of abandoned CPSs, before which it is a prerequisite to understand the behaviors of Cr, especially for its migration and distribution pattern in the soils of CPSs.

The migration and speciation transformation of Cr in soils will be influenced by many factors, such as the valence state of Cr ions, the soil pH and Eh values, the soil organic matter concentration, and the soil manganese dioxide concentration [5,6]. Cr(III) mainly exists in a cation form (e.g., CrCl_3_ and Cr(NO_3_)_3_), and it is prone to combine with organic ligands in soil [7]. Cr(VI) mainly exists in an anion form (e.g., HCrO^4-^, Cr_2_O_7_^2−^, and CrO_4_^2−^), and it can migrate easily in soil [8]. When the soil pH < 6, Cr(III) possesses a high migration capability; conversely, when the soil pH > 6, it is Cr(VI) in anion form that possesses high migration capability [9]. The Cr(III) in soil is capable of transforming into Cr(VI) quickly under the influence of oxides, especially manganese dioxide [10]. In contrast, the Cr(VI) in soil can be reduced to Cr(III) by Fe^2+^, S^2−^, and soil organic matter under anaerobic conditions [11].

Previous studies have provided a scientific basis for elucidating the distribution of Cr pollution, and a few research studies indicated that the distance to the pollutant source and the landfill of COPR will affect the horizontal and vertical distribution of Cr in CPSs, respectively [12,13,14]. However, studies on the horizontal and vertical distribution patterns and influencing factors are still needed to guide the investigation and the risk assessment of CPSs.

Lots of chromate production plants in the world were constructed along the river in general, and the soil in these sites always contaminated by improper disposal of COPR. The CPS of Changsha, which is located nearby the Xiangjiang River, covered an area of approximately 76,000 m^2^, and the soil was contaminated by improper dispose of COPR as well. Therefore, the studies on the distribution patterns of Cr were conducted in the CPS of Changsha to achieve the following objectives: (1) to uncover the horizontal and vertical distribution patterns of Cr in a CPS based on chemical and statistical analysis; and, (2) to investigate the factors affecting the horizontal and vertical distributions and migration patterns of Cr in a CPS.

## 2. Materials and Methods

### 2.1. Study Area

**Figure 1:**
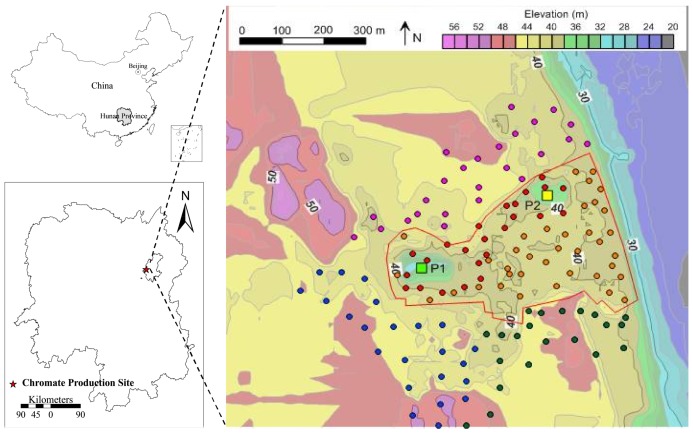
Location of the CPS. Red Line: boundary of the chromate production sites; Red Dots: soil profiles of the chromite ore processing residues (COPR) Storage Area; Orange Dots: soil profiles in the Production Plant Area; Pink Dots: soil profiles in the Xiangyue Chemical Plant; Green Dots: soil profiles in the Residential District; Blue Dots: soil profiles in the Changsha Zinc Plant; Square: remaining slag pits after dealing with COPR. The green and yellow squares were designated as P1 and P2, respectively.

The studied CPS is located in the valley of an industrial park in Changsha City, Hunan Province, China (28°15′49″–28°16′08″ N, 112°57′10″–112°57′20″ E). Two pits remained in the CPS after the removal of 42 thousand tons of COPR: P1 (depth = 14 m) and P2 (depth = 5 m). The Xiangyue chemical plant, Changsha zinc plant, and a residential district are located near the CPS (NA-CPS) (Figure 1). The average elevations of the COPR storage area, production plant area, Xiangyue chemical plant, Changsha zinc plant, and residential district are 40 m, 40 m, 43 m, 45 m, and 46 m, respectively. The average elevation of NA-CPS is 3–6 m higher than that of the CPS (Figure 1).

The soils (0–14 m) in the CPS have four layers, including miscellaneous fills, clayey soil, medium–fine sand, and a gravel layer. The miscellaneous fills in the COPR storage area comprise clayey soils, COPR, and construction waste, but the soil in the production plant area does not contain COPR. Water injection and penetration tests were conducted to determine the permeability coefficient of the different soil layers. The permeability coefficient of the miscellaneous fills, clay, medium–fine sand, and gravel are 15 m/day, 0.086 m/day, 17 m/day, and 40 m/day, respectively.

The phreatic water in the medium–fine sand and gravel layers has an uneven distribution without a continuous and stable water surface during the dry season, and the water level is from 6.8–14.1 m at the CPS (elevation: 26.75–36.35 m). The lowest water level (31 October 2009), highest flood level (28 June 1998), and the annual mean water level of the Xiangjiang River is 24.87 m, 39.18 m, and 29.48 m, respectively (Wusong elevation). The wet season extends from April to September, and the groundwater consumption period often occurs within the period of October to the following March.

### 2.2. Soil Sampling and Analysis

The burial depth of phreatic water in the CPS is up to 14 m, and the soil below the phreatic water (14 m) mostly consists of weathered rock formations. Therefore, 61 soil profiles (depth: 14 m) were excavated by Geoprobe (Model: 6620 DT; Pipe Diameter: 5 cm) on a grid system at intervals of 35 min throughout the CPS (24 soil profiles in the COPR storage area and 37 soil profiles in the production plant area). Overall, 732 soil samples were collected at depths of 0.0–0.2, 0.2–0.6, 0.6–1.2, 1.2–2.0, 2.0–3.0, 3.0–4.0, 4.0–5.0, 5.0–6.0, 6.0–8.0, 8.0–10.0, 10.0–12.0, and 12.0–14.0 m in the CPS. In the Xiangyue chemical plant, Changsha zinc plant, and residential district, 25, 21, and 23 soil samples, respectively, were excavated at depths of 0.3–0.5 m. In addition, According to The Technical Specification for soil Environmental monitoring (HJ/T166-2004), two background soil profiles were excavated at each of the three woodland, which are 1–1.5 km north, west, and south of CPS, respectively. Overall, 24 background soil samples were collected at depths of 0.3–0.5, 3.0–3.2, 6.0–6.2, and 12.0–12.2 m. The perched (2.0–8.0 m) and phreatic water (13.0–16.0 m) were sampled from ten profiles in the CPS for testing Cr and Cr(VI) concentrations. The geographic coordinate system (GCS_Beijing_1954) and elevation for each sampling point were recorded by an RTK (Zhong Haida V30, Guangzhou, China). The burial depths of the different soil layers (including miscellaneous fills, clays, medium–fine sand, and gravels) were all recorded.

The soil samples were air-dried and sieved through a 2-mm polyethylene sieve. A portion of each soil sample (50 g) was ground in an agate grinder and sieved through a 100 mesh-sieve in preparation for total Cr analysis. The soil samples were digested in groups of four with a mixture of HNO_3_, HCl, HF, and HClO_4_ [15]. In order to assess the leaching toxicity of Cr polluted soil and the toxicity and migration ability of Cr in the leachate under the weak acid rainfall process, the Solid waste—Extraction procedure for leaching toxicity—Sulphuric acid & nitric acid method (SES) was used [16]. The total Cr concentrations in the SES leachate (*L_Cr_*), groundwater, and the digestion solution were determined by Inductively Coupled Plasma Optical Emission Spectrometry (Thermo iCAP 6300, Shanghai, China). The Cr(VI) in the SES leachate (*L_Cr_*^6+^) and groundwater were determined by ultraviolet visible spectrophotometer (TU-1810, Beijing, China). Soil pH was measured in distilled water at a soil solution ratio of 1:2.5 (*w*/*v*) using a Mettler Toledo 320 pH meter. All of the chemicals used were of guaranteed reagent grade. Blanks, 10% duplicated samples, and soil standard reference materials (Centre for Reference Materials, Beijing, China) were analyzed as part of quality assurance and quality control (QA/QC) procedures. The analyses of the recoveries for the various elements were within the range of 95–103%.

### 2.3. Calculations of Distance, LR_Cr_, and P_Cr_^6+^ and the Elevations of Sampling Points

The GCS_Beijing_1954 coordinates of the central points of P1 (X = 46,411.135, Y = 106,782.06) and P2 (X = 46,586.4, Y = 106,897.71) were recorded. The distances of one profile to the central points of P1 and P2 were calculated, and the distance with the smaller value was retained for subsequent analysis:

(1) $$\begin{array}{cc} \mathrm{Distance}=\mathrm{Min} & (\sqrt{{\left(x_{i}-46411.14\right)}^{2}+{\left(y_{i}-106782.06\right)}^{2}}\\ & :\sqrt{{\left(x_{i}-46586.40\right)}^{2}+{\left(y_{i}-106897.71\right)}^{2}}) \end{array}$$

where Distance refers to the minimum distance of one profile to the central points of P1 and P2, and *x_i_* and *y_i_* refer to the GCS_Beijing_1954 coordinates.

The leaching rate of soil Cr (*LR_Cr_*) can be used to describe the ratio of the easy-to-migrate Cr to the soil Cr concentration, reflecting the migratory aptitude of Cr in the soil. The percentage of Cr(VI) to total Cr in the leachate (*P_Cr_*^6+^) reflects the toxicity and migration ability of Cr in the leachate, because Cr(VI) is a carcinogenic substance that is recognized by the World Health Organization, and Cr(VI) possesses higher migration capability than Cr(III) under alkaline conditions:

(2) $$LR_{Cr}=\frac{L_{Cr}\times 10}{C_{Cr}}\times 100\%$$

where *LR_Cr_* refers to the leaching rate of total Cr in the soils of the CPS and *L_Cr_* refers to the total Cr concentration in the SES leachate; 10 refers to the liquid-solid ratio, according to SES, and *C_Cr_* refers to the total Cr concentration in the soils.

(3) $$P_{Cr^{6+}}=\frac{L_{Cr^{6+}}}{L_{Cr}}\times 100\%$$

where *P_Cr_*^6+^ refers to the percentage of Cr(VI) to total Cr in the SES leachate, *L_Cr_*^6+^ refers to the concentration of Cr(VI) in the SES leachate, and *L_Cr_* refers to the total Cr concentration in the SES leachate.

### 2.4. Longitudinal Distribution of Cr Content in Soils

Linear, exponential, and binomial distribution functions were used to fit the Cr concentration to the depth for each profile in the CPS and the NA-CPS using SAS software (SAS Inst., Cary, NC, USA). The top coordinates ($-\frac{b}{2a}$, $-\frac{4ac-b^{2}}{4a}$) of the binomial distribution functions were calculated for each soil profile in the CPS. The Cr concentration of the soil profiles obeys a positive binomial distribution (P-BD) when the parameter *a* is greater than zero. The Cr concentration decreases with increasing depth in the range of (0, $-\frac{b}{2a}$) m and increases with an increasing depth in the range of ($-\frac{b}{2a}$, 14) m. The Cr concentration of the soil profiles obeys a negative binomial distribution (N-BD) when the parameter *a* is smaller than zero. Finally, the Cr concentration increases with an increasing depth in the range of (0, $-\frac{b}{2a}$) m and decreases with an increasing depth in the range of ($-\frac{b}{2a}$, 14) m.

(4) $$C_{\mathrm{lg}Cr}=ad+b,$$

(5) $$C_{\mathrm{lg}Cr}=a\exp^{bd}+b,$$

(6) $$C_{\mathrm{lg}Cr}=ad^{2}+bd+c,$$

where *C*_lg*Cr*_ refers to the Cr concentration after a logarithmic transformation, *d* refers to the depth of each sample central point location, and *a*, *b*, and *c* are parameters with respect to the different distribution functions.

The predicted values of the Cr concentration were back-calculated, according to the parameters (i.e., *a*, *b*, and *c*) and depth. The values of *R*^2^ and the root mean square error (*RMSE*) between the predicted and observed values of the Cr concentrations were calculated to assess the goodness of fit and the uncertainty of the predictions. Larger *R*^2^ values reflect better suitability of the fitting function, and smaller values of *RMSE* indicate the greater reliability of the predictions:

(7) $$RMSE=\sqrt{\frac{1}{n}\sum_{i=1}^{n}{\left[{\overset{⌢}{C}}_{\mathrm{lg}Cr}(d_{i})-C_{\mathrm{lg}Cr}(d_{i})\right]}^{2}}$$

where ${\overset{⌢}{C}}_{\mathrm{lg}Cr}(d_{i})$ refers to the predicted value of the Cr concentration in soils, $C_{\mathrm{lg}Cr}(d_{i})$ refers to the observed value of the Cr concentration in soils, and *n* refers to the number of observed values.

### 2.5. Statistical Analysis

Microsoft Office’s Excel 2010 (Microsoft Office 2010, Microsoft Corporation, Redmond, WA, USA) was used to calculate the mean values, coefficients of variation, and standard deviations. Here, a variance analysis was conducted for the data that obeyed a normal distribution. A nonparametric test was conducted for the data that disobeyed a normal distribution (Kruskal–Wallis and Mann–Whitney U). A Pearson correlation analysis and equation regression analysis were conducted using SPSS16.0 software (SPSS16.0, International Business Machines Corporation, Chicago, IL, USA) for the parameters that included elevation, distance, pH, Cr concentration, *L_Cr_*, *L_Cr_*^6+^, *LR_Cr_*, and *P_Cr_^6+^*. In regression analysis, soil Cr concentration in soil was lg-transformed to ensure homogeneity of variances. In addition, there are no significant differences among the different soil depths for the background values of pH, Cr concentration, *L_Cr_*, and *L_Cr_*^6+^. Thus, the background value indicates an average value.

## 3. Results

### 3.1. General Characteristics of Surface Soils

**Figure 2:**
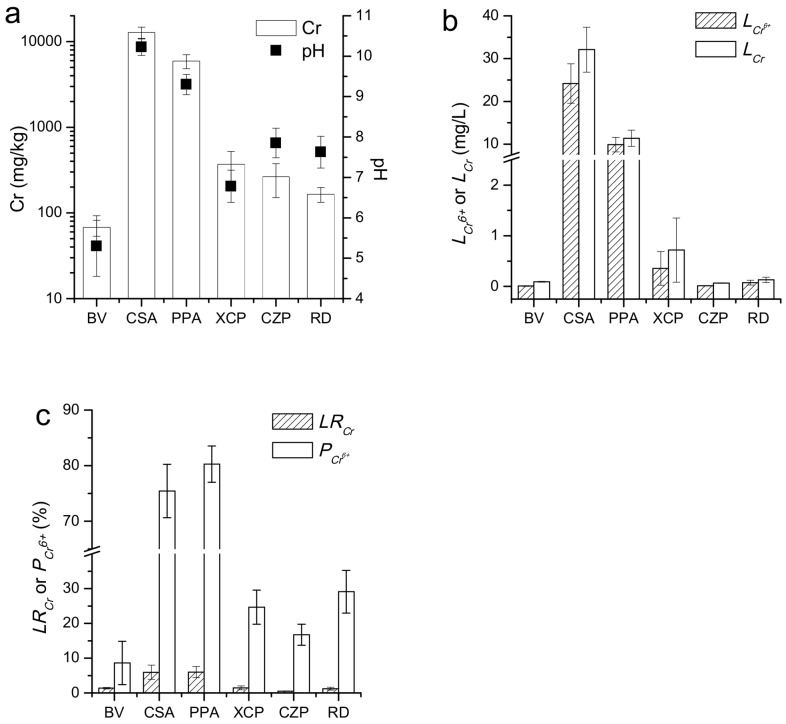
The Cr concentration and pH (**a**), *L_Cr_* and *L_Cr_*^6+^ (**b**), *LR_Cr_* and *P_Cr_^6+^* (**c**) of the surface soils. BV: Background Values; CSA: COPR Storage Area; PPA: Production Plant Area; XCP: Xiangyue Chemical Plant; CZP: Changsha Zinc Plant; RD: residential district.

The average pH of the surface soils in the COPR storage area is 0.9 units higher than in the production plant area and 2.4–3.4 units higher than in the three NA-CPS areas (Xiangyue chemical plant, Changsha zinc plant, and the residential district). In addition, the pH of the NA-CPS is 1.5–2.6 units higher than the background value (pH = 5.3) (Figure 2a). Similarly, the Cr concentrations, *L_Cr_*, *L_Cr_*^6+^, *P_Cr_^6+^*, and *LR_Cr_* of the surface soils in the CPS are significantly higher than in the NA-CPS (*p* < 0.01) (Figure 2a–c). Moreover, the Cr concentrations, *L_Cr_*, *L_Cr_*^6+^, and *P_Cr_*^6*+*^ of the surface soils in the three NA-CPS areas are similar: 2.4–5.4, 0.8–7.8, 1.5–45, and 2.0–3.4 times greater than the respective background value (Cr = 67.8 mg/kg, *L_Cr_* = 0.092 mg/L, *L_Cr_*^6+^ = 0.008 mg/L, *P_Cr_*^6*+*^ = 8.6%). The average values of the *LR_Cr_* of the surface soils in the three NA-CPS areas are similar to the background value (*LR_Cr_* = 1.36%). The Cr concentration, pH, *L_Cr_*, *L_Cr_*^6+^, *LR_Cr_* and *P_Cr_^6+^* of background soils have been showed as Table A1.

### 3.2. Distribution of Cr Content in Surface Soils

Linear function fitting, exponential function fitting, and multiple linear stepwise regression analysis indicate that the distribution of the Cr concentration in surface soils is related to distance, elevation, and pH (Table 1). The *R*^2^_adj_ values of the linear fitting equation for the Cr concentration to distance, elevation, and pH are 0.374, 0.315, and 0.451 (*p* < 0.001), respectively. The *R*^2^_adj_ values of the exponential fitting equation for the Cr concentration to distance, elevation, and pH are 0.351, 0.319, and 0.460 (*p* < 0.001), respectively. There is no obvious difference between the linear fitting equation and the exponential fitting equation in terms of *R*^2^_adj_. Furthermore, the multiple linear regression function, including elevation, distance, and pH, accounts for 63.2% of the variance (*R*^2^_adj_ = 0.632), and it shows predictions that are more accurate than the single-factor regression analysis.

### 3.3. Distribution of the Cr Concentration in Soil Profiles

The vertical distribution of the Cr concentration in the CPS is simulated using linear, exponential, and binomial functions. The *R*^2^ values (*R*^2^ = 0.743) of the binomial distribution functions are higher than those of the linear and exponential functions. Furthermore, the *RMSE* values (*RMSE* = 0.293) of the binomial distribution function are smaller than those of the linear and exponential functions (Table 2). This indicates that the Cr concentrations of the soil profiles in the study area mainly obey a binomial distribution, rather than an exponential or linear distribution. Further analysis indicates that the vertical distributions of Cr in the 51 soil profiles (accounting for 84% of the total profiles) of the CPS obey a binomial distribution. Twenty-nine and 22 soil profiles obey P-BD (*R*^2^ = 0.218, *p* < 0.001) and N-BD (*R*^2^ = 0.208, *p* < 0.001), respectively. Parameters *a*, *b*, and *c* for the binomial distributions (P-BD and N-BD), averaged at 0.016 ± 0.012 and −0.007 ± 0.006, –0.268 ± 0.133 and 0.032 ± 0.085, and 3.973 ± 0.597 and 3.862 ± 0.467, respectively. The value of $-\frac{b}{2a}$ for P-BD averaged at 8.4 ± 2.2, which indicates that the Cr concentration decreases with an increasing depth in the range of 0–8 m and increases with an increasing depth in the range of 8–14 m. The value of $-\frac{b}{2a}$ for N-BD is smaller than zero, which indicates that the Cr concentration decreases with increasing depth in the range of 0–14 m (Table 3).

### 3.4. General Characteristics of Soil Profiles Obeying a Binomial Distribution

**Figure 3:**
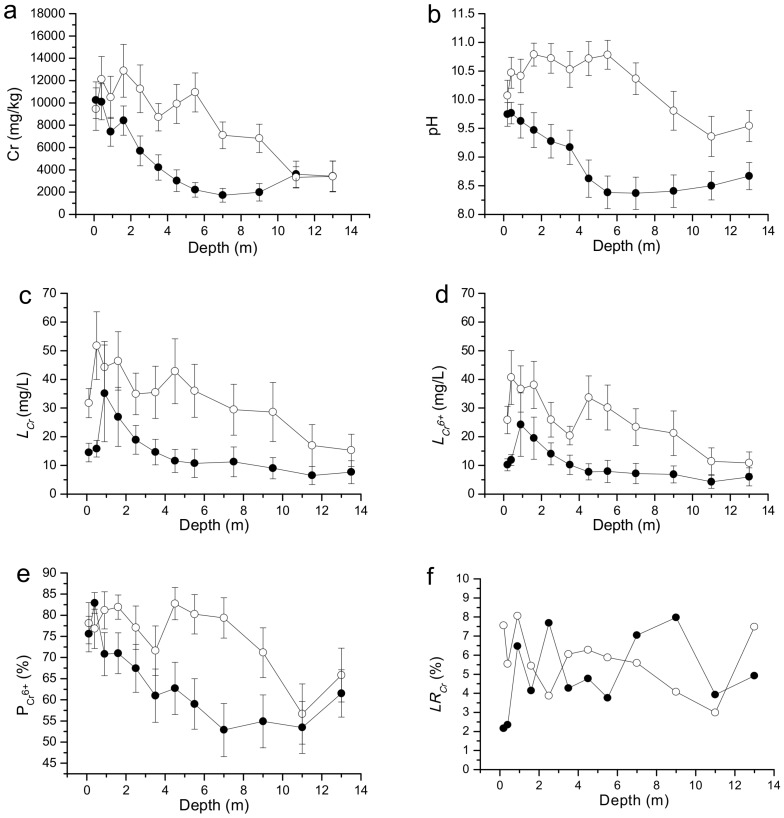
The Cr concentration (**a**), pH (**b**), *L_Cr_* (**c**), *L_Cr_*^6+^ (**d**), *LR_Cr_* (**e**) and *P_Cr_^6+^* (**f**) of soil profiles that obey binomial distribution changes with depth in the chromate production sites (CPS). Solid Circle: soil profiles obey a Positive Binomial Distribution (*n* = 29); Open Circle: soil profiles obey a Negative Binomial Distribution (*n* = 22).

As can be seen in Figure 3a, for the soil profiles in the CPS that obey the P-BD, the average Cr concentrations in the soil of the six layers at 0.0–2.0, 2.0–4.0, 4.0–6.0, 6.0–8.0, 8.0–10.0, and 10.0–14.0 m are 9044 ± 1468, 4958 ± 1238, 2613 ± 811, 1719 ± 623, 1984 ± 788, and 3518 ± 1262 mg/kg, respectively. The Cr concentration in the soil profiles decreases significantly with increasing depth in the range of 0.0–8.0 m (*p* < 0.01), but it shows an upward trend with increasing depth in the range 8.0–14.0 m. For the soil profiles in the CPS that obey N-BD, the average Cr concentrations in three soil layers at 0.0–6.0, 6.0–10.0, and 10.0–14.0 m are 10721 ± 1884, 6960 ± 1230, and 3367 ± 1166 mg/kg, respectively. The Cr concentration is stable in the soil layer of 0.0–6.0 m, and it decreases clearly with an increasing depth in the range of 6.0–14.0 m. In addition, there is no significant difference in the Cr concentrations in the layers of 0.0–2.0 m and 10.0–14.0 m between the soil profiles that obey the P-BD and N-BD in the CPS (*p* > 0.05). However, the Cr concentrations in the layer of 2.0–10.0 m in the soil profiles of the CPS that obey the P-BD are significantly lower than in the equivalent layer of the soil profiles that obey the N-BD (*p* < 0.01).

As can be seen in Figure 3b–f, the pH, *L_Cr_*, *L_Cr_*^6+^, and *P_Cr_*^6+^ of the soil profiles that obey the P-BD in the CPS decrease significantly with an increasing depth in the range of 1.2–5.0 m (*p* < 0.01), but they show no significant differences in the range of 5.0–14.0 m. To the contrary, the pH, *L_Cr_*, *L_Cr_*^6+^, and *P_Cr_*^6+^ of the soil profiles in the CPS that obey the N-BD show no significant differences in the range of 0.0–5.0 m (*p* > 0.05), but they decrease significantly with an increasing depth in the range 5.0–12.0 m. The values of pH, *L_Cr_*, *L_Cr_*^6+^, and *P_Cr_*^6+^ of the soil profiles in the CPS that obey the N-BD are significantly higher than in the profiles that obey the P-BD (*p* < 0.01). The above analysis indicates that the pH, *L_Cr_*, *L_Cr_*^6+^, and *P_Cr_*^6+^ of the soil profiles in the CPS show similar vertical distributions to Cr. However, the *LR_C_*_r_ of the soil profiles in the CPS shows no significant difference with increasing depth in the range of 0.0–14.0 m.

### 3.5. General Characteristics of Different Soil Layers

**Figure 4:**
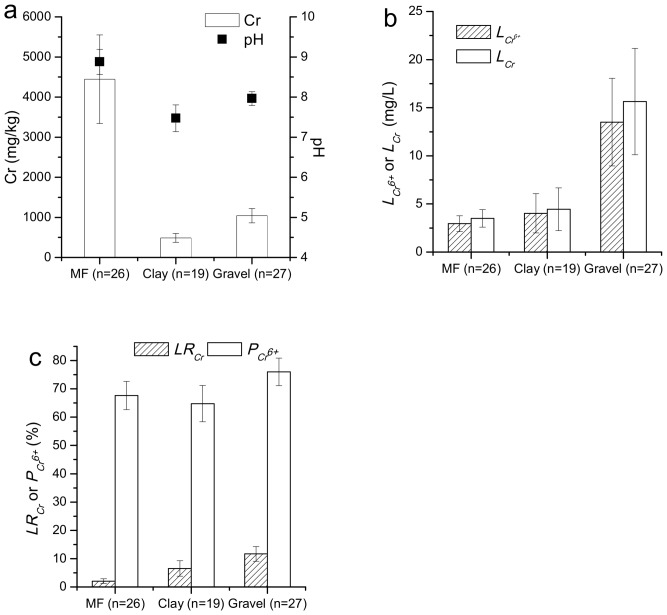
The Cr concentration and pH (**a**), *L_Cr_* and *L_Cr_*^6+^ (**b**), *LR_Cr_* and *P_Cr_^6+^* (**c**) of the different soil layers. MF: Miscellaneous Fill.

In order to investigate the influence of the soil layer on the migration capability of Cr in the soil profiles, eight profiles with clay layers in the production plant area were selected to test the Cr concentration, pH, *L_Cr_*, and *L_Cr_*^6+^. The Cr concentrations and the pH in the miscellaneous fill are significantly higher than those in the gravel and clay (*p* < 0.01) (Figure 4a). However, the *L_Cr_* and *L_Cr_*^6+^ of the miscellaneous fill and the clay are significantly lower than that of the gravel (*p* < 0.01) (Figure 4b). Similar to *L_Cr_* and *L_Cr_*^6+^, the *LR_Cr_* values of the miscellaneous fill and the clay are also significantly lower than those of the gravel (*p* < 0.01) (Figure 4c). Therefore, the type of soil layer has a significant impact on the *LR_Cr_* in soil profiles.

## 4. Discussion

### 4.1. Horizontal Distribution of Cr in Soil

The distribution pattern of the Cr concentration in the surface soils of the study area can be represented in terms of a multiple linear regression equation (lgCr = 4.436 + 0.217 × pH − 0.003 × distance − 0.07 × elevation, *R*^2^_adj_ = 0.632). The COPR is the outcome of the chromate producing process, using an excessive amount of calcium oxide, sodium carbonate, and chromic iron ores via high-temperature roasting [17]. As a result, the pH of the COPR leachate was as high as 10–12, and the Cr concentration in the COPR reached 46 g/kg [18,19]. Over 90% of the total Cr exists in the form of Cr^6+^, when the pH of the leachate of COPR is above eight [20]. Therefore, the migration capability of Cr usually increases with a rise in pH [21]. This is evident by the fact that the pH values of the surface soils have extremely significant positive correlations with *P_Cr_*^6+^ (*R* = 0.520) and Cr concentration (*R* = 0.526) (Table 4). As a result, soil pH could affect the distribution of the Cr concentration by increasing its migration capability. On the one hand, clay minerals, metallic oxides, and organic substances in soils are able to adsorb heavy metals that migrate in association with surface runoff [22]. On the other hand, the OH^−^ and Cr in the COPR storage area and producing plant area cannot migrate to the surface soils of the NA-CPS via surface runoff, because of the effect of elevation. Therefore, the Cr concentrations of the surface soils have a significantly negative correlation with elevation and distance (*R* = −0.523 and −0.402, respectively) (Table 4). A research study conducted in the CPS of Qing Hai indicated that the relationship of the Cr concentration with the distance to COPR can be fitted by the linear function or power function, and the power function is better than the linear function [13]. In addition, the Cr concentrations of the surface soils in the NA-CPS are significantly higher than the background values, indicating that the Cr-bearing flying dust might affect the distribution of Cr in the surface soils of the NA-CPS. Heavy metals migration with flying dust also occurred from the activities of mining and smelting in the Pb-Zn mine [23,24]. In summary, pH, distance, and elevation are the primary factors that influence the distribution of Cr concentration in the surface soils.

### 4.2. Vertical Distribution of Cr in Soil

**Figure A1:**
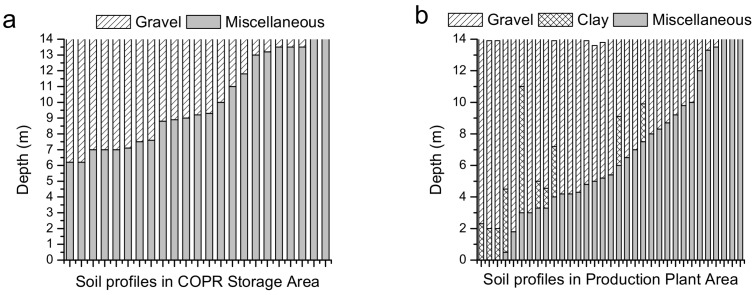
Soil layer structures of the soil profiles at 0–14 m in COPR Storage Area (**a**) and Prouduction Plant Area (**b**).

Twenty-nine soil profiles (*y* = 0.013*d*^2^ − 0.026*d* + 3.805) in the CPS obey the P-BD. The Cr concentrations of the profiles that obey the P-BD decrease significantly with an increasing depth in the range of 0.0–8.0 m, but increase with increasing depth in the range of 8.0–14.0 m. Although at least 90% of Cr(III) in the soil solution can be fixed by soil [25], only a few parts of Cr(VI) in the soil solution will be fixed by soil [26,27]. However, in the process of permeating to the subsoil with rainwater, the Cr(VI) can be reduced into Cr(III) by sulfides, ferrous iron, and soil organic matter under the condition of relatively low pH and Eh (−1.37 V < Eh < −1.35 V) [28,29]. Therefore, the Cr concentration decreases with increasing depth in the range of 0.0–8.0 m, because of the reduction and fixing effect of the soil. In addition, the values of parameter *b* for P-BD have significant positive correlations with *L_Cr_*, *L_Cr_^6+^*, and *LR_Cr_* at depths of 3–14 m (*R* = 0.35–0.48), and the values of parameter *c* for P-BD has significant positive correlations with pH and the concentration of Cr (*R* = 0.35–0.91) at depths of 0–10 m (Table 5). These results indicate that the Cr concentration, pH, and *LR_Cr_* of different layers of soil will affect the decreasing amplitude of the Cr concentration at depths of 0–10 m. A research study conducted on the vertical migration of Cr in a CPS of Chongqing City shows that the Cr content decreases with an increasing depth at ranges of 0–16.0 m at the producing plant area, COPR transfer site, and Living office area [14]. The Cr concentration of phreatic water in the CPS averaged 218 mg/L, and the soil layer at depths of 10.0–14.0 m in the CPS is mainly composed of gravel (Figure A1), which is conducive to the migration of Cr-polluted phreatic water [30]. As a result, the soil at depths of 10.0–14.0 m is contaminated by the Cr-polluted phreatic water. This can be proved by the fact that the Cr concentration of the soil at depths of 10.0–14.0 m is significantly higher than at 6.0–10.0 m (*p* < 0.01). A similar result has been shown by a research study on the southern area of the CPS in Chongqing City under a railway bridge in which the landfill of COPR has caused the Cr content to increase with increasing depth at ranges of 4.0–16.0 m [14]. In summary, the Cr concentrations in the soil profiles obey the P-BD because of the fixing effect of the soil and the influence of Cr-polluted phreatic water, and the decreasing amplitude of the Cr concentration for the P-BD is mainly affected by the Cr concentration, pH, and *LR_Cr_* of the soil.

Twenty-two soil profiles (*y* = −0.006*x*^2^ + 0.027*x* + 3.829) in the CPS obey the N-BD. The Cr concentration shows a stable level at soil layers at 0.0–6.0 m, and it decreases obviously with increasing depth at ranges of 6.0–14.0 m. This shows that the Cr in the subsoil at depths of 0.0–6.0 m comes from not only the topsoil, but also other pollutant sources, such as COPR in the miscellaneous fill. The soil layer at 0.0–6.0 m in the COPR storage area mainly comprises miscellaneous fills mixed with COPR (Figure A1a), and the COPR can elevate the Cr concentration of the surrounding soil [31]. As a result, no significant difference was found for the Cr concentration in the soil layer at 0.0–6.0 m. The Cr concentration in the soil layer at 10.0–14.0 m is derived not only from the Cr of the upper layer soil, but also from the Cr in the phreatic water. However, this combined effect is insufficient to elevate the Cr concentration in the soil layer at 10.0–14.0 m to the levels similar to or higher than the soil layer at 8.0–10.0 m (Figure 3a). The values of parameter *b* for N-BD have significant positive correlations with *L_Cr_*, *L_Cr_^6+^*, and *LR_Cr_* at depths of 4–14 m (*R* = 0.47–0.62); the values of parameter *c* for N-BD have positive correlations with pH and the concentration of Cr (*R* = 0.19–0.92) at depths of 0–10 m, and they have negative correlations with *LR_Cr_* (*R* = −0.07–−0.75) at depths of 0–14 m (Table 5). These results indicate that the Cr concentration, pH, and *LR_Cr_* in different layers of soil will affect the decreasing amplitude of the Cr concentration at depths of 0–14 m. In summary, the Cr concentrations in the soil profiles obey the N-BD, mainly because of the influence of miscellaneous fills containing COPR and the fixing effect of the soil, and, similar to the results from the P-BD, the decreasing amplitude of the Cr concentration for N-BD is also mainly affected by the Cr concentration, pH, and *LR_Cr_* of the soil.

As discussed above, OH^−^ and Cr share the same polluted resource, and the *P_Cr_*^6+^ usually increases with a rise in pH. In addition, the *P_Cr_*^6+^, *L_Cr_*, and *L_Cr_*^6+^ have significant positive correlations with the Cr concentration (*R* = 0.501–0.654) (Table 4). Therefore, the pH, *L_Cr_*, *L_Cr_*^6+^, and *P_Cr_*^6+^ of the soil profiles in the CPS show similar vertical distributions to the Cr concentration. The *LR_C_*_r_ of the soil profiles in the CPS shows no significant difference with increasing depth at ranges of 0.0–14.0 m. This should be attributed to the fact that the *LR_Cr_* in soil profiles is mainly affected by the type of soil layer, rather than the Cr concentration. The fine grains in the miscellaneous fill and clay possess higher surface activity and larger surface areas, and they comprise a greater proportion of the substance that serves to enhance the adsorption capacity of the soils [22]. As a result, the *LR_Cr_* values of the miscellaneous fill and the clay are significantly lower than that of the gravel. Previous research has found that the vertical migration distance and the transmission rate of heavy metals in sandstone soils are far higher than in clay soils [30]. In summary, the *P_Cr_*^6*+*^ of soil increases with pH, and the type of soil layer is the primary factor that influences the *LR_Cr_* in the soil profiles. 

## 5. Conclusions

The studied CPS was seriously contaminated by Cr, and the NA-CPS was also contaminated by Cr. The migration of Cr in the surface soils of the study area can be represented in terms of a multiple linear regression equation (*R*^2^_adj_ = 0.632). Distance, elevation, and pH are the primary factors that influence the horizontal distribution of the Cr concentration in the surface soils.

The subsoil (up to 14 m) and groundwater in the CPS were also seriously contaminated by Cr. A binomial distribution function is suitable for representing the vertical distributions of Cr in the soil profiles of the study area. The Cr concentrations in the soil profiles obey the P-BD because of the fixing effect of the soil and the influence of Cr-polluted phreatic water. The Cr concentrations in the soil profiles that obey the N-BD exist mainly because of the influence of miscellaneous fills containing COPR and the fixing effect of the soil. The decreasing amplitude of the Cr concentration for binomial distributions is mainly affected by the Cr concentration, pH, and the leaching rate of Cr in the soil. The pH, *L_Cr_^6+^*, and *P_Cr_^6+^* of the soil profiles show similar vertical distributions to the Cr concentration. The migration capability of Cr in soil profiles is mainly affected by pH and soil type. Our results suggest that the horizontal and vertical distributions of Cr in a chromate production district could be used to guide the investigation and risk assessment of a CPS.

## Figures and Tables

**Table 1:** Fitted simple linear and exponential regressions of the value of the Cr concentration after lg-transformation (lgCr) to pH, distance, or elevation, and a summary of the multiple linear correlations between lgCr in soil and pH, distance, and elevation in the form of *y* = *a* + *bx*_1_ + *cx*_2_ + *dx*_3_ (stepwise regression. Criteria: probability of F to enter *p* ≤ 0.050, probability of F to remove *p* ≥ 0.100).

Equation	*R*	*R*^2^_adj_	*p*
lgCr = 3.934 − 0.008 × distance	0.616	0.374	<0.001
lgCr = 3.927 × exp(−0.003 × distance)	0.598	0.353	<0.001
lgCr = 9.403 − 0.155 × elevation	0.567	0.315	<0.001
lgCr = 27.482 × exp(−0.055 × elevation)	0.570	0.319	<0.001
lgCr = 0.311 + 0.298 × pH	0.675	0.451	<0.001
lgCr = 1.102 × exp(0.106 × pH)	0.681	0.460	<0.001
lgCr = 4.436 + 0.217 × pH − 0.003 × distance − 0.07 × elevation	0.800	0.632	<0.001

Chromium concentration in soil was lg-transformed to ensure homogeneity of variances.

**Table 2:** The *R*^2^ and root mean square error (*RMSE*) values for different distribution functions.

Functions	Equation	*R*^2^	*R*^2^_adj_	*RMSE*
Linear Function	Predicted = 1.197 + 0.649 lgCr	0.649	0.648	0.320
Exponential Function	Predicted = 1.162 + 0.659 lgCr	0.656	0.655	0.320
Binomial Distribution Function	Predicted = 0.878 + 0.743 lgCr	0.743	0.742	0.293

Chromium concentration in soil was lg-transformed to ensure homogeneity of variances.

**Table 3:** Fitted binomial regressions of lgCr to depth (*d*) in the form of *y* = *ax*^2^ + *bx* + *c*).

Binomial Type	Positive Binomial Distribution (*n* = 29)	Negative Binomial Distribution (*n* = 22)
*a*	0.016 ± 0.012	−0.007 ± 0.006
*b*	−0.268 ± 0.133	0.032 ± 0.085
*c*	3.973 ± 0.597	3.862 ± 0.467
($-\frac{b}{2a}$, $-\frac{4ac-b^{2}}{4a}$)	(8.4 ± 2.2, 2.7 ± 0.4)	(−2.0 ± 4.4, 4.1 ± 0.4)
equations	lgCr = 0.013 × d^2^ − 0.026 × d + 3.805	lgCr = −0.006 × d^2^ + 0.027 × d + 3.829
*R*^2^	0.218	0.208
*p*	<0.001	<0.001

Chromium concentration in soil was lg-transformed to ensure homogeneity of variances.

**Table 4:** Pearson correlation coefficients among general characteristics of surface soils in the study area (*n* = 130).

	Elevation	Distance	pH	Cr	*L_Cr_*^6+^	*L_Cr_*	*P_Cr_*^6+^
Distance	0.607 **						
pH	−0.303 **	−0.394 **					
Cr	−0.402 **	−0.523 **	0.526 **				
*L_Cr_*^6+^	−0.322 **	−0.454 **	0.485 **	0.642 **			
*L_Cr_*	−0.351 **	−0.489 **	0.504 **	0.654 **	0.965 **		
*P_Cr_*^6+^	−0.515 **	−0.499 **	0.520 **	0.501 **	0.525 **	0.466 **	
*LR_Cr_*	−0.257 **	−0.160	0.100	−0.090	0.282 **	0.273 **	0.356 **

** Correlation is significant at the 0.01 level (two-tailed).

**Table 5:** The correlation coefficient of parameters to the factors of pH, lgCr, *L_Cr_*, *L_Cr_*^6+^, *P_Cr_*^6+^, and *LR_Cr_* in the different depths of soil.

Parameter	Depth (m)	P-BD (*n* = 29)						N-BD (*n* = 22)					
		pH	*L_Cr_*^6+^	*L_Cr_*	*P_Cr_^6+^*	*LR_Cr_*	lgCr	pH	*L_Cr_*^6+^	*L_Cr_*	*P_Cr_^6+^*	*LR_Cr_*	lgCr
*b*	0.0–0.2	0.07	0.15	0.16	−0.12	0.32	−0.35	−0.16	−0.45 *	−0.48 *	0.01	0.28	−0.38
	0.2–0.6	−0.14	−0.15	−0.07	−0.07	0.22	−0.43 *	−0.04	−0.23	−0.21	0.07	0.58 **	−0.60 **
	0.6–1.2	−0.18	0.30	0.29	−0.06	0.34	−0.3	0.02	−0.45 *	−0.41 *	−0.26	−0.16	−0.53 *
	1.2–2.0	−0.07	0.25	0.26	−0.10	0.31	−0.44 *	−0.20	−0.18	−0.09	−0.52 *	0.65 **	−0.63 **
	2.0–3.0	−0.13	0.22	0.24	0.02	0.27	−0.14	−0.37	0.12	0.07	−0.01	0.49 *	−0.34
	3.0–4.0	−0.13	0.37 *	0.34	0.26	0.47 *	0.04	−0.3	0.18	0.39	−0.09	0.54 *	−0.13
	4.0–5.0	−0.09	0.40 *	0.40 *	0.23	0.48 *	0.06	−0.26	0.58 **	0.57 **	0.10	0.56 **	−0.14
	5.0–6.0	−0.01	0.39 *	0.38 *	0.59 **	0.48 *	0.28	−0.27	0.58 **	0.62 **	−0.38	0.37	−0.03
	6.0–8.0	−0.02	0.41 *	0.40 *	0.38 *	0.33	0.21	0.16	0.47 *	0.48 *	−0.42	0.26	0.10
	8.0–10.0	−0.33	0.42 *	0.43 *	0.11	0.33	−0.10	0.21	0.56 **	0.53 *	0.06	0.62 **	−0.13
	10.0–12.0	−0.24	0.35 *	0.38 *	−0.02	0.35 *	−0.02	0.26	0.47 *	0.47 *	0.41	0.30	0.09
	12.0–14.0	−0.28	0.38 *	0.38 *	0.26	0.39 *	−0.02	−0.17	0.58 **	0.57 **	0.40	0.56 **	0.06
*c*	0.0–0.2	0.35 *	0.41 *	0.28	0.56 **	−0.08	0.69 **	0.47 *	0.57 **	0.63 **	−0.11	−0.36	0.54 *
	0.2–0.6	0.59 **	0.47 *	0.42 *	0.07	−0.03	0.88 **	0.46 *	0.44 *	0.45 *	−0.27	−0.70 **	0.92 **
	0.6–1.2	0.57 **	0.13	0.12	0.56 **	−0.05	0.91 **	0.44 *	0.62 **	0.62 **	0.02	−0.07	0.80 **
	1.2–2.0	0.50 **	0.08	0.06	0.30	−0.03	0.85 **	0.67 **	0.40	0.37	0.34	−0.64 **	0.84**
	2.0–3.0	0.71 **	0.29	0.28	0.34	0.09	0.73 **	0.80 **	0.25	0.35	−0.14	−0.60 **	0.82 **
	3.0–4.0	0.73 **	0.19	0.19	−0.06	−0.23	0.60 **	0.71 **	−0.07	−0.03	−0.13	−0.74 **	0.71 **
	4.0–5.0	0.55 **	0.06	0.04	−0.03	−0.13	0.56 **	0.63 **	−0.30	−0.23	−0.13	−0.75 **	0.60 **
	5.0–6.0	0.56 **	0.01	0.01	−0.01	−0.13	0.43 *	0.64 **	−0.52 *	−0.58 **	0.26	−0.74 **	0.57 **
	6.0–8.0	0.53 **	0.01	0.01	−0.13	−0.04	0.19	0.31	−0.15	−0.15	0.20	−0.28	0.27
	8.0–10.0	0.59 **	−0.05	−0.04	0.02	−0.09	0.30	0.01	−0.30	−0.27	0.17	−0.60 **	0.45 *
	10.0–12.0	0.38 *	−0.06	−0.03	−0.12	−0.27	0.41 *	−0.07	−0.17	−0.15	−0.25	−0.10	0.15
	12.0–14.0	0.32	−0.07	−0.08	−0.25	−0.15	0.03	0.31	−0.38	−0.29	−0.41	−0.51 *	0.25

* Correlation is significant at the 0.05 level (two-tailed); ** Correlation is significant at the 0.01 level (two-tailed).

## Appendix A

**Table A1:** General characteristics of background soils.

Depth (m)	pH	Cr (mg/kg)	*L_Cr_*^6*+*^ (mg/L)	*L_Cr_* (mg/L)	*LR_Cr_* (%)	*P_Cr_*^6*+*^ (%)
0.3–0.5	5.15 ± 0.7	68.7 ± 11.6	0.008 ± 0.006	0.102 ± 0.008	1.52 ± 0.29	8.4 ± 6.6
3–3.2	5.35 ± 0.8	64.0 ± 11.3	0.009 ± 0.009	0.094 ± 0.011	1.35 ± 0.04	9.1 ± 9.7
6–6.2	5.46 ± 1.1	71.6 ± 5.2	0.006 ± 0.003	0.088 ± 0.010	1.17 ± 0.10	6.6 ± 3.1
12–12.2	5.32 ± 0.4	66.8 ± 22.6	0.010 ± 0.004	0.085 ± 0.005	1.38 ± 0.45	10.3 ± 5.5
Average values	5.30 ± 0.75	67.8 ± 14.4	0.008 ± 0.006	0.092 ± 0.010	1.36 ± 0.22	8.6 ± 6.24
